# The Giant HECT E3 Ubiquitin Ligase HERC1 Is Aberrantly Expressed in Myeloid Related Disorders and It Is a Novel BCR-ABL1 Binding Partner

**DOI:** 10.3390/cancers13020341

**Published:** 2021-01-19

**Authors:** Muhammad Shahzad Ali, Cristina Panuzzo, Chiara Calabrese, Alessandro Maglione, Rocco Piazza, Daniela Cilloni, Giuseppe Saglio, Barbara Pergolizzi, Enrico Bracco

**Affiliations:** 1 Department of Clinical and Biological Science, Medical School, University of Torino, 10043 Orbassano, Italy; muhammad.ali79@edu.unito.it (M.S.A.); cristina.panuzzo@unito.it (C.P.); chiara.calabrese@unito.it (C.C.); alessandro.maglione@unito.it (A.M.); daniela.cilloni@unito.it (D.C.); giuseppe.saglio@unito.it (G.S.); 2 Department of Health Sciences, University of Milano-Bicocca, 20900 Monza, Italy; rocco.piazza@unimib.it; 3 Department of Oncology, Medical School, University of Torino, 10043 Orbassano, Italy

**Keywords:** E3 ubiquitin ligases, ubiquitin proteasome system, myeloid neoplasms, *Bcr-Abl1*, *HERC1*, gene expression, protein–protein interaction

## Abstract

**Simple Summary:**

The pathological role/s of the HERC family members has recently been initiated to be explored in few solid tumors and the assessment of their transcript amount reveals that they might act as effective prognostic factors. However, evidence concerning the non-solid tumors, and especially myeloid related neoplasms, is currently lacking. In the present article for the first time we provide original data for a clear and well-defined association between the gene expression level of a giant HERC E3 ubiquitin ligase family member, HERC1, and some myeloid related disorders, namely Acute Myeloid Leukemia, Myeloproliferative neoplasms and Chronic Myeloid Leukemia. Furthermore, our findings unveil that the HERC1 protein physically interacts, likely forming a very large supramolecular complex, and it is a putative BCR-ABL1 tyrosine kinase substrate. We hope that this work will contribute to the advance of our understanding of the roles played by the giant HERCs in myeloid related neoplasms.

**Abstract:**

HERC E3 subfamily members are parts of the E3 ubiquitin ligases and key players for a wide range of cellular functions. Though the involvement of the Ubiquitin Proteasome System in blood disorders has been broadly studied, so far the role of large HERCs in this context remains unexplored. In the present study we examined the expression of the large HECT E3 Ubiquitin Ligase, HERC1, in blood disorders. Our findings revealed that *HERC1* gene expression was severely downregulated both in acute and in chronic myelogenous leukemia at diagnosis, while it is restored after complete remission achievement. Instead, in Philadelphia the negative myeloproliferative neoplasm *HERC1* level was peculiarly controlled, being very low in Primary Myelofibrosis and significantly upregulated in those Essential Thrombocytemia specimens harboring the mutation in the calreticulin gene. Remarkably, in CML cells *HERC1* mRNA level was associated with the BCR-ABL1 kinase activity and the HERC1 protein physically interacted with BCR-ABL1. Furthermore, we found that HERC1 was directly tyrosine phosphorylated by the ABL kinase. Overall and for the first time, we provide original evidence on the potential tumor-suppressing or -promoting properties, depending on the context, of *HERC1* in myeloid related blood disorders.

## 1. Introduction

The function and activity of almost every cellular protein can be modulated by posttranslational modifications (PTMs) [1]. In particular, ubiquitination has emerged as one of the most conserved eukaryotic PTMs from yeast to human [2]. The ubiquitination is a reversible and energy-dependent enzymatic process by which the ubiquitin is either covalently linked to substrate proteins or alternatively can form free polyubiquitin chains. The ubiquitination process requires three classes of enzymes, namely ubiquitin activating (E1), ubiquitin conjugating (E2) and ubiquitin ligase (E3). The ubiquitin ligases are the terminal enzymes transferring the ubiquitin to the e-amino group of a lysine residue on the substrate or the amino-terminus of a polypeptide [3]. The E3 ligases often function as multi-subunit complexes determining the substrate specificity [4]. In humans, the E3 ligase family comprises more than 600 members classified into three sub-families, based on their structural and catalytic features, named RING (Really Interesting New Gene), HECT (Homologous to the E6AP Carboxyl Terminus) and RBR (Ring Between Ring). The HECT and RBR catalyse the ubiquitin transfer to the substrate by forming an E3-ubiquitin thioester intermediate while RING members do not possess direct catalytic activity but rely on the enzymatic activity of E2 to ubiquitinate the substrate [5].

The E3 ubiquitin ligases regulate many biological processes through timely ubiquitination and degradation of many cellular proteins responsible for gene transcription/repression, receptor endocytosis, intracellular trafficking, signal transduction, cell cycle progression, DNA damage recognition and repair, autophagic clearance, differentiation and apoptosis [6,7]. For these reasons, dysregulation of E3 ligases often leads to the development of several forms of diseases such as central nervous system disorders, inflammation, metabolic dysfunctions and several cancers [8,9] including hematologic malignancies [10]. Remarkably, several ubiquitin ligases are mutated, overexpressed or deleted in the hematologic malignancies, contributing to the pathogenesis of the diseases through accumulation, or excessive degradation, of their substrates that may in turn acquire critical functions during the hematopoietic cell growth and differentiation [10]. Deletions within the locus of the large HECT E3 ubiquitin ligase HERC1 have been observed in acute lymphoblastic leukemia (ALL) and have been associated to an increased resistance to thiopurine antileukemic agents due to the control that HERC1 exerts on the stability of the DNA mismatch repair enzyme MSH2 [11]. In addition, *HERC1* point mutations were recurrently found in few leukemias such as T-ALL [12], AML [13] and T-cell prolymphocytic leukemia [14]. Eventually, a very rare and atypical HERC1-PML transcript fusion mRNA was reported in acute promyelocytic leukemia [15]. Besides these reports, in which the HERC1 mutational status has been associated with different leukemia, the cellular functions of this protein in leukemia cells have been scarcely investigated. Thanks to the efforts made by the Human Protein Atlas Project (proteinatlas.org) it has emerged that the differential gene expression of HERC1 members might act as prognostic factor in few solid tumours.

Indeed, recent findings indicate that HERC1 finely controls the ERK signaling by regulating the c-Raf stability. HERC1 knockdown rises p38 activity by increasing the levels of MKK3, an MAPKK, in a manner dependent on C-RAF [16]. Additionally, we lately identified a large *Dictyostelium* HECT member (HectPH1), exhibiting many structural similarities with the mammalian HERC1, as a suppressor of the mTORC2-PKB/AKT signaling axis [17]. 

Hence, in the present study we sought to explore the role of *HERC1* in myeloid blood related disorders, to assess its potential role in the leukemogenesis process. Our findings revealed that *HERC1* gene expression was severely down-regulated both in acute and in chronic myelogenous leukemia while it is peculiarly modulated in the myeloproliferative neoplasms, suggesting that in myeloid malignancies HERC1 display either tumor-suppressing and -promoting properties depending on the context. Remarkably, in CML cells the *HERC1* transcript amount is associated to the BCR-ABL1 kinase activity and HERC1 protein interacted with BCR-ABL1. Eventually, we showed that HERC1 is tyrosine phosphorylated by the Abl kinase. Overall and for the first time, we provide original evidence on the potential role of *HERC1* in myeloid related blood disorders.

## 2. Results

### 2.1. HERC1 Gene Expression Is Aberrantly Regulated in Myeloid Related Disorders and Leukemias

**Figure 1:**
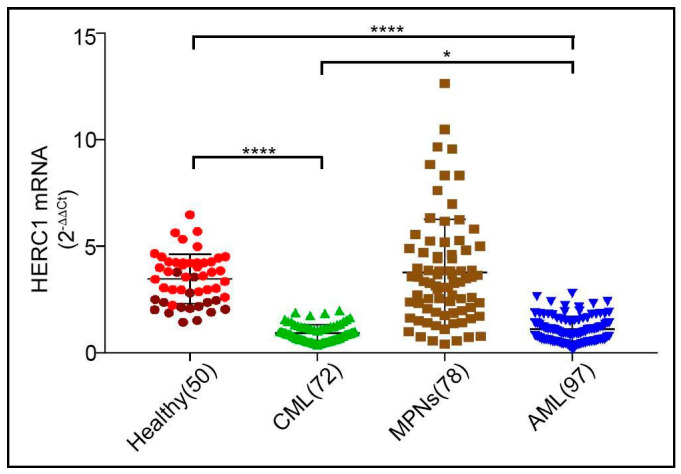
The *HERC1* gene expression is differently regulated in bone marrow (BM) and peripheral blood (PB) and it is dysregulated in haematological malignancies. *HERC1* transcript was assayed in healthy donors including both PB (red dots) and BM (dark red dots), and in a panel of myeloid related disorders at diagnosis, namely Chronic Myeloid Leukemia (CML) (PB = 37, BM = 35), Acute Myeloid Leukemia (AML) (PB = 17, BM = 80), MyeloProliferative Neoplasms (MPNs) (PB = 75) by RT-qPCR. The *HERC1* mRNA quantity was expressed as 2^−ΔΔCt^ after normalization against *GUSB*. HERC1 gene expression was significantly downregulated in newly diagnosed CML and AML, with median values of 0.9 and 1 respectively, when compared to healthy specimens (median = 4.01). In MPNs the HERC1 expression fluctuated from 0.4 to 12.65 with a median value of 3.3 and thus not significantly different from healthy subjects. *p*-values were calculated on the differences in *HERC1* gene expression observed between patients and the healthy control specimens. * *p* < 0.05, **** *p* < 0.0001.

Despite the mutational status of the genes, encoding for the large HERCs has been scrutinized in different myeloid and lymphoid blood tumors [11,12,14], but their expression levels in normal Bone Marrow (BM) and Peripheral Blood (PB) from healthy subjects has never been assessed. Hence, initially we determined the *HERC1* gene expression by 2^−ΔΔct^ methods in a cohort of healthy subjects by comparing its mRNA abundance in PB and BM specimens. Interestingly, PB and BM displayed different levels of *HERC1* mRNA, with the amount in PB being significantly higher than in BM (Figure S1). Indeed, PB showed values that were approximately twice those found in BM (PB median = 4.01 vs BM median = 2.20) (Figure 1, red and dark red dots, respectively). Since our interest was related to myeloid related disorders, we then assessed the *HERC1* gene expression in a panel of myeloid malignancies including AML (*n* = 97), MPNs (*n* = 78.) and CML (*n* = 72).

When compared to the pool of PB and BM healthy specimens, the *HERC1* gene expression was sharply downregulated at diagnosis both in acute (median value approximately of 1.0) and in chronic myeloid (median value approximately 0.93) leukemia. Differently, in MPNs specimens we observed that the *HERC1* gene expression level did not significantly differ from that of the healthy subjects, albeit the values appeared rather disperse within a window ranging from 0.40 to 12.65 with a median value of 3.33 (Figure 1). On the whole, *HERC1* gene expression is differentially regulated in PB and in BM, markedly downregulated both in AML and CML and expressed with a rather scattered pattern in the MPNs group.

### 2.2. Newly Diagnosed AML Display HERC1 Down-Regulation Independently from the Genetic Alterations

**Figure 2:**
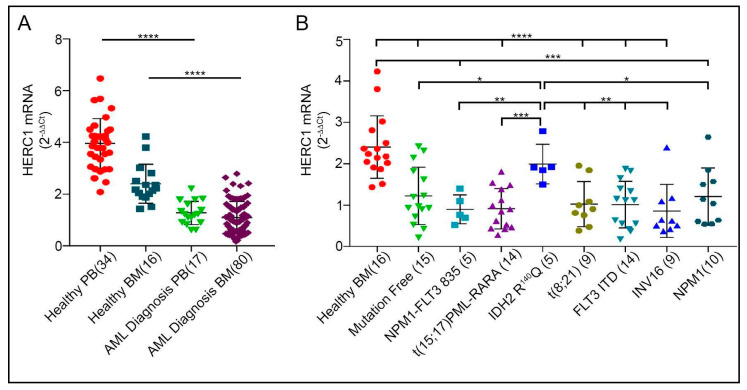
The *HERC1* transcript is severely downmodulated in acute myeloid leukemia independently from genetic alteration. (**A**) *HERC1* level is significantly downregulated in both PB (*n* = 17) and BM (*n* = 80) specimens derived from AML at diagnosis. (**B**) The stratification of the AML samples according to the different cytogenetic categories and to the most common genetic variants displayed that the *HERC1* transcript downregulation is a common feature shared by almost all the different groups with the exception of the *IDH2* mutated cohort, in which its gene expression is comparable to that of the controls. * *p* < 0.05, ** *p* < 0.01, *** *p* < 0.001, **** *p* < 0.0001.

The AML is the most common type of leukemia in adults and is characterized by a high degree of genetic heterogeneity, including inactivation of oncosuppressors [18]. Hence, it is among the most challenging myeloid neoplasms to be treated [19]. We examined the gene expression of *HERC1* in this type of leukemia according to the most common cytogenetic and genetic alterations. The *HERC1* expression level was assessed in newly diagnosed AML (80 were BM and 17 PB specimens). Interestingly, both the BM and the PB specimens collected from newly diagnosed AML patients exhibited comparable *HERC1* transcript amounts (BM median = 0.97 vs PB median = 1.12), revealing that the expression levels were distinctly decreased in both BM and PB patient’s samples (*p* < 0.0001) (Figure 2A).

Since AML displays quite a broad genetic diversity we stratified the BM specimens collected at diagnosis according to the most common cytogenetic and genetic variants, being the resulting groups comparatively age-matched (Supplementary Table S4). By comparing the *HERC1* transcript among the different categories we did not observe any major difference (Figure 2B) besides the *IDH2* mutated specimens, in which the *HERC1* gene expression was significantly higher when compared to all the other AML subgroups (ANOVA, *p* = 0.03), displaying a median level (1.9) similar to that of the healthy donors (*p* = 0.27).

These results indicated that in AML patients the *HERC1* gene expression level was significantly lower at diagnosis and not associated with any of the different cytogenetic/genetic group with the interesting exception of the five AML patients harboring the IDH2 R140Q mutation which displayed *HERC1* levels indistinguishable from the control group.

### 2.3. MPNs Display a Peculiar Pattern of HERC1 mRNA Abundance

Philadelphia chromosome-negative myeloproliferative neoplasms (MPNs) are hematopoietic stem-cell disorders characterized by an abnormal proliferation of one or more terminal myeloid cells. They include Essential Thrombocythemia (ET), Polycythemia Vera (PV) and Primary Myelofibrosis (PMF). The PV and ET are chronic diseases associated with a long life expectancy, while PMF is a more aggressive disease characterized by harsher symptoms. As opposed to the Philadendia-positive myeloid neoplasm, namely CML, the MPNs are characterized by different somatic genetic mutations involving *JAK2*, Calreticulin (CALR) and the thrombopoietin receptor (MPL) encoding genes [20].

**Figure 3:**
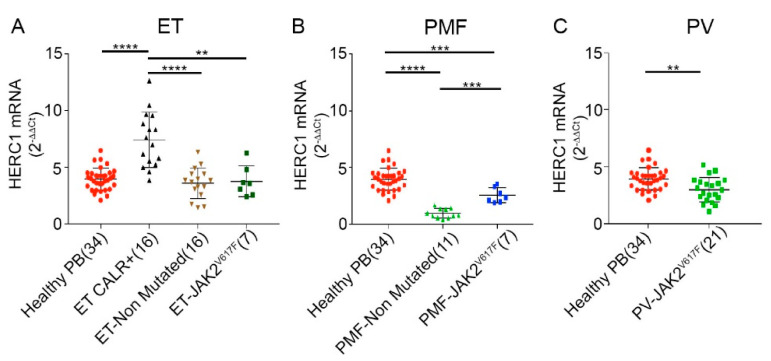
*HERC1* mRNA is differentially and peculiarly expressed in MPNs patients. On the whole, the expression pattern of *HERC1* in MPNs appeared to be rather scattered and variable. Among MPNs, when compared to the healthy subjects, the CALR mutated Essential Thrombocythemia (ET) specimens (**A**) displayed a significantly higher amount of HERC1 transcript, (*p* ≤ 0.0001). On the contrary, the *JAK2* mutated, and non-mutated, specimens showed levels comparable to the control counterpart. (**B**) Differently, the Primary Myelofibrosis (PMF) patients displayed a rather steep downregulation of *HERC1* gene expression. However, the JAK2V617F mutated samples showed higher *HERC1* levels in comparison to the mutation free cohort. (**C**) In Polycythemia Vera (PV) patients the *HERC1* mRNA level was slightly, but significantly, lower than that of the healthy subjects (** *p* < 0.01, *** *p* < 0.001, **** *p* < 0.0001).

The expression pattern of *HERC1* in MPNs appeared rather scattered and different in each category. Among the three different MPNs, the PB samples from ET patients (*n* = 39) were those displaying the most variable *HERC1* gene expression levels (ranging from 1.4 to 12.6). Interestingly, while either the ET specimens harboring the JAK2V617F mutation or those non-mutated displayed *HERC1* levels similar to the controls, those mutated for CALR exhibited significantly higher *HERC1* mRNA level (median 7.3) (Figure 3A). Conversely, the non-mutated PMF patients (*n* = 11) displayed a very low amount of *HERC1* transcript (median = 0.8) (Figure 3B) with values very similar to those previously observed in AML and CML specimens. Worthy of note, the PMF JAK2V617F mutated patients showed a slightly, but significantly (*p* = 0.0001) higher level of *HERC1* mRNA (median = 2.3) when compared to the non-mutated group. Eventually, specimens from Jak2 V617F PV patients showed a moderate, though significant, lowering in *HERC1* gene expression level (Figure 3C).

### 2.4. The HERC1 Expression Is Sensitive to the Bcr-Abl1 Tyrosine Kinase Activity

**Figure 4:**
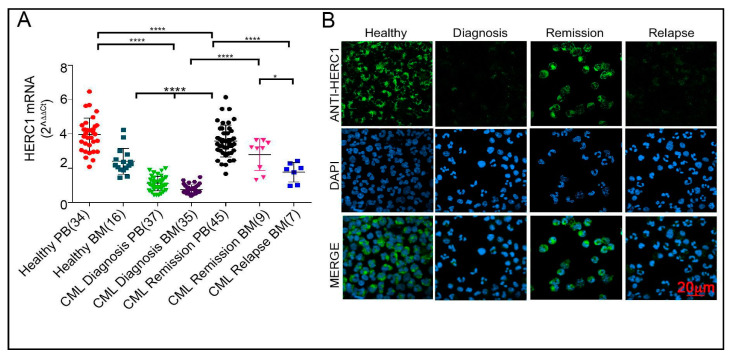
*HERC1* gene expression is differently regulated in CML patients during the evolution of the disease. (**A**) *HERC1* gene was differentially expressed during the different CML phases being severely down-regulated at diagnosis, both in BM and PB CML specimens, increased at remission and declined again at the onset of relapse. (**B**) Similarly to the mRNA, in primary CML cells the HERC1 protein amount was barely detectable at diagnosis and at the relapse onset. On the contrary, at remission the protein level was comparable to that of healthy control. Immunofluorescence was performed on cytospun cells by using specific rabbit polyclonal anti HERC1 antibody. DAPI staining (blue) indicated the cell’s nuclei. The green signal corresponds to the HERC1 protein. * *p* < 0.05, **** *p* < 0.0001.

By measuring the transcript level of *HERC1* in CML patients at diagnosis we noticed that this was decreased in both PB (median = 1.03) and BM (median = 0.66) specimens. We then asked whether *HERC1* expression might be affected by the different CML clinical stages, including diagnosis, remission and relapse. Our analysis unveiled that after the molecular remission (BCR-ABL1^IS^ ≤ 0.1%) the level of the *HERC1* transcript sharply increased reaching values (median = 3.5) comparable to those observed in the control subjects (median = 3.5 and 3.1 for PB and BM, respectively) (Figure 4A). Ultimately, when patients relapsed again a reduction in *HERC1* gene expression occurred. Due to the very large size of the HERC1 protein, in human primary cells we encountered technical difficulties in blotting it. Hence, we decided to determine the HERC1 protein levels in CML specimens collected at different clinical stages (diagnosis, remission and relapse) by immunofluorescence. Consistently with gene expression results, the HERC1 protein amount was low at diagnosis but increased during remission and declined again when patients relapsed (Figure 4B).

**Figure 5:**
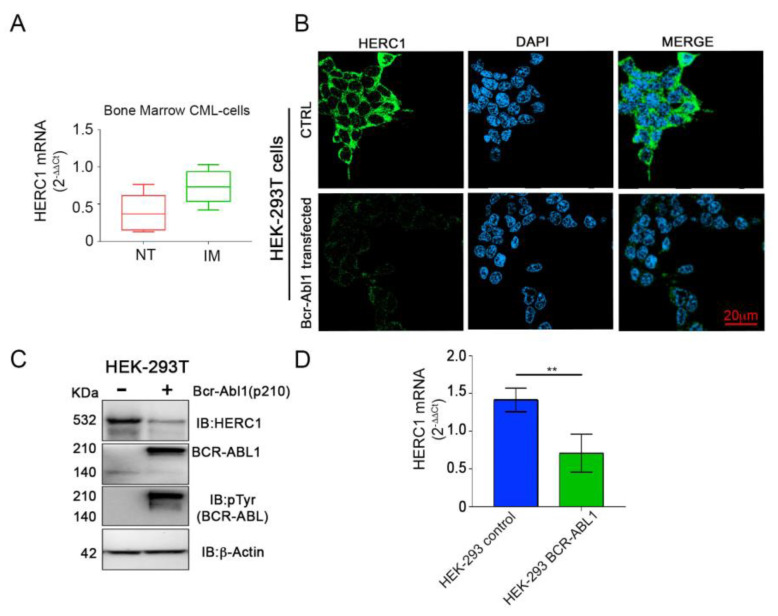
HERC1 mRNA and protein amounts are regulated by the Bcr-Abl1 tyrosine kinase activity. (**A**) Primary bone marrow derived from leukemic cells of newly diagnosed CML patients (*n* = 5) displayed a modest increase in HERC1 mRNA quantity after in vitro Imatinib (2.5 µM) treatment. (**B**,**C**) HEK-293T transiently and exogenously expressing the constitutively active p210 BCR-ABL1 tyrosine kinase exhibited a severe down-modulation of HERC1 protein examined by immunofluorescence (**B**) and Western Blot (**C**). DAPI staining (blue) indicated the cells nuclei while the green signal was corresponding to HERC1 protein. (**D**) *HERC1* mRNA level was significantly reduced in HEK-293T overexpressing the BCR-ABL1 (** *p* < 0.01).

In CML patients, the remission is usually achieved in response to tyrosine kinase inhibitors (e.g., Imatinib^®^) monotherapy. Therefore, we assessed the HERC1 expression at both mRNA and protein levels in primary CML cells treated for 48 h with Imatinib. Interestingly, Imatinib-dependent BCR-ABL1 inhibition enhanced, though not significantly, the *HERC1* transcript amount (Figure 5A).

These results prompted us to ascertain whether the BCR-ABL1 tyrosine kinase might affect the *HERC1* expression. Hence, we transfected the HEK-293T cell line with the p210 *BCR_ABL1* overexpressing plasmid. Interestingly, the *BCR-ABL1* overexpression led to a significant lowering of HERC1 both at transcript and protein level (Figure 5B–D) in agreement with the findings in CML specimens at diagnosis.

### 2.5. HERC1 Is a Novel Binding Partner and a Substrate of BCR-ABL1

Besides the HECT and the RLD domains, and despite their very large size, the HERC E3 Ub-ligases harbor only few recognizable structural regions [21]. Noticeably, many somatic mutations associated with different tumors fall within unknown structural constrained amino acid stretches [22]. In the last years quite a few efforts have been made to identify HERCs interacting partners with the aim of gaining insight of the cellular functions of these proteins [23]. While currently the HERC2 accounts for dozens of binding partners [24], no more than a bunch have been experimentally ascertained for HERC1.

**Figure 6:**
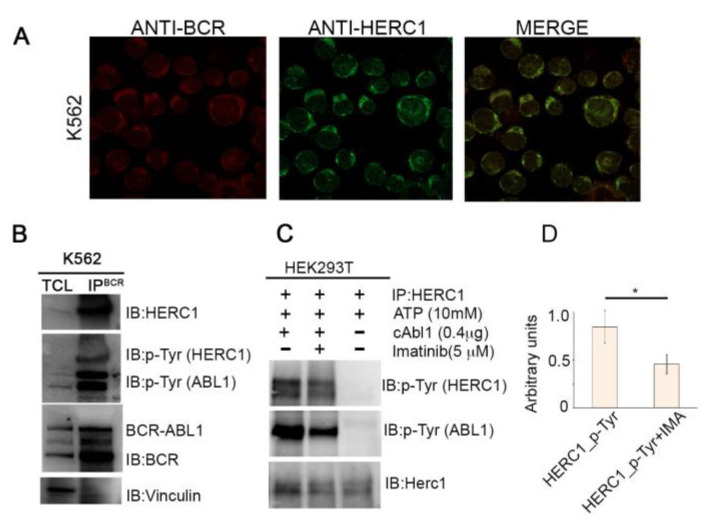
HERC1 is a novel interactor and a potential substrate of Bcr-Abl1. (**A**) The subcellular localization of either the HERC1 or Bcr-Abl1 (p210) proteins were assessed by staining cytospun K562 cells with anti-HERC1 and anti-Bcr specific antibodies. The immunofluorescence assay revealed that the localization of the two proteins was mostly restricted to the cytoplasmic compartment and they co-localized. (**B**) In K562 cells BCR-ABL1 and HERC1 proteins form a complex. The immunoprecipitated BCR-ABL1 was employed to pull-down HERC1 from K562 total cell lysate. The presence of HERC1 in the immunoprecipitated sample was determined by immunoblotting the membrane with HERC1 antibody (upper panel). Subsequently probing with a tyrosine phospho-specific antibody proved the phosphorylated HERC1 status. Vinculin was used as loading control (lower panel). (**C**) In vitro kinase assay with purified c-ABL and HERC1 substrate, obtained after HERC1 immunoprecipitation on 293T, performed in the absence or presence of 5 µM Imatinib treatment for 30 min. (**D**) The effect of the Imatinib showed in (**C**) was measured quantitatively using the ImageJ software. The bands intensity, corresponding to HERC1-P-Tyr protein, were quantified and normalized to the amount of HERC1 protein signal for each lane (the max value was set at 1). Normalized arbitrary units of three different experiments with SD are shown in the bar graphs. * *p* < 0.05.

By analyzing and comparing the HERC1 and BCR-ABL1 subcellular localization in K-562 cells, we observed that both proteins displayed a diffuse pattern and mostly they co-localized (Figure 6A). It is noteworthy that HERC1 showed a more spotted pattern, when compared to BCR-ABL1, with “*speckle-like*” structures mainly dispersed within the cytoplasmic compartment. These observations prompted us to investigate whether the two proteins might also interact.

To this purpose, we attempted to pull-down HERC1, in K562 Ph-positive cells, by co-immunoprecipitation experiment using as bait BCR-ABL. As revealed by the intense and sharp HERC1 Western Blot band, obtained after BCR-ABL1 immunoprecipitation, the two proteins not only colocalized but they also interacted with each other (Figure 6B).

Interestingly, the HERC1 turns out to be even tyrosine phosphorilated (Figure 6B). Since BCR-ABL1 displays constitutive tyrosine kinase activity and phosphorylates a very broad spectrum of different effectors, we next asked whether the HERC1 tyrosine phosphorylation status might be affected by BCR-ABL1 itself. Hence, we determined the HERC1 tyrosine phosphorylation status in K562 cells. Immunoprecipitation of K562 lysates with an anti-phosphotyrosine specific antibody and the subsequent immunoblotting with anti-HERC1 antibody unveiled that HERC1 is one among many proteins heavily phosphorylated in this CML cell line (Figure S2A). An in-silico search (www.cbs.dtu.dk/services/NetPhos) revealed that HERC1 protein harbors several putative tyrosine phosphorylation sites and at least three of them (Y905, Y3867 and Y4452) appeared to be *bona fide* BCR-ABL1 phosphorylation consensus motif [25]. Altogether, these bodies of evidence suggested that BCR-ABL1 may directly phosphorylate HERC1. To validate this surmise, we performed an “in-vitro” kinase assay by mixing the purified c-ABL1 kinase with the putative HERC1 substrate. c-ABL1 directly phosphorylated HERC1 on tyrosine residues, and this phosphorylation was significantly inhibited by treatment with Imatinib (Figure 6C,D). On the whole these findings indicate that HERC1 is a novel binding partner and a substrate of BCR-ABL1.

## 3. Discussion

To overcome stresses and to properly balance the entire proteome, every eukaryotic cell relies on the ubiquitin proteasome system (UPS). Damaged, or malfunctioning, proteins are ubiquitin tagged and then degraded by the 26S proteasome. However, the ubiquitination process is not exclusively engaged under stress conditions. Indeed, ubiquitin moieties can serve as a docking site for protein–protein interaction. Eventually, as important as the ubiquitin conjugates the cell survival and proliferation rely also on the free ubiquitin chains and the free ubiquitin pool [26]. Alterations in the UPS are often associated with severe diseases and numerous E3 Ubiquitin ligases are aberrantly regulated in different cancers [27,28].

Despite the HECT sub-family members representing a small proportion of the E3 ubiquitin ligase superfamily many of them have been found to be either aberrantly regulated or mutated in several tumors [8,9]. Among HECT proteins, the HERC subfamily comprises two giant members, HERC1 and HERC2. Mutations in either genes have been reported in some kinds of lymphoid leukemia (e.g., acute lymphoblastic and pro-lymphocytic leukemia) and solid tumors (e.g., breast cancers, non-melanoma skin and gastric carcinoma) [22].

However, currently, specific inquiries aiming to assess the potential role of the giant HERCs in myeloid related neoplasia are lacking. We initially started by appraising the *HERC1* transcript amount in healthy subjects and by comparing its levels between the PB and the BM. Afterwards, we moved to a panel of myeloid related disorders including AML, MPNs and CML. Interestingly, our findings revealed that in healthy control specimens the *HERC1* is differentially transcribed when comparing PB and BM, displaying significantly lower levels in the BM (Figure S1). Furthermore, by determining the quantitative expression of the *HERC1* gene in a panel of myeloid related disorders we found a steep decline in those samples collected from patients with newly diagnosed AML, CML and PMF (Figure 1). Noteworthy, alike *HERC1*, the ARIH2 gene that encodes for an RBR member named TRIAD1 is expressed at a very low level in BM but it steeply rises in PB. Though the *ARIH2* transcript amount in acute promyelocytic cell line (NB4) is low [29], suggesting a similar expression pattern in acute myeloid leukemia patients, an inclusive study aiming to evaluate its expression level in a cohort of patients affected by different myeloid disorders is lacking. On the contrary, the molecular mechanisms controlling the *ARIH2* gene expression, and its cellular role in regulating the hematopoiesis, have been comprehensively scrutinized in cell lines and murine models [30,31]. Overall, these studies unveiled that TRIAD1 inhibits the clonogenic growth of primary myeloid progenitor cells but it is required to induce both granulocytic and monocytic differentiation thus making it a player in myeloid cell differentiation [29]. Based on our findings we cannot exclude that, similarly to TRIAD1, HERC1 might be involved in blood cell differentiation thus contributing to the proper hematopoiesis. Future investigations, aiming to functionally explore the functional role of HERC1 in blood cells, are required. Akin to HERC1, the WWP1 is a member of the HECT family and its clinical relevance in AML has been assessed. Contrarily to *HERC1* the WWP1 expression is significantly upregulated in AML specimens. Nonetheless, as observed for *HERC1* the WWP1 transcript levels are regulated independently from the different cytogenetic categories and genetic variants [32]. Remarkably, we additionally perceived that those patients harboring the IDH2-R140Q mutation showed higher *HERC1* transcript levels when compared to all the other cytogenetic categories and genetic variants we screened (Figure 2B). The mutated IDH2 enzyme is characterized by a peculiar neomorphic activity resultant in the synthesis of a novel oncometabolite, the 2-hydroxyglutarate, which in turn inhibits the α-ketoglutarate-dependent epigenetic regulators (e.g., histone demethylase TETs). As a consequence, the genomic DNA results hypermethylated thus contributing to profoundly impair the cellular differentiation [18,33]. Our results advise to argue that, likely, the IDH2-R140Q-dependent genomic DNA hypermethylation leads to the transcriptional repression of an, by now, unknown transcriptional repressor and thus maintains unaltered the *HERC1* mRNA levels. Further research will be required to ascertain this hypothesis.

Conversely from PMF, the other two MPNs showed higher *HERC1* levels that were either quite similar to those of the healthy subjects, as it occurred for PV, or even higher as it was in the case of the *CALR* mutated ET samples (Figure 3A). Unambiguously, our findings indicate that in MPNs the *HERC1* transcript inversely correlates with the severity of the disease, the PMF specimens being those with the lowest level of *HERC1*, but not with the JAK2 mutational status in PV and ET. Furthermore, among MPNs the CALR mutated ET patients, displaying a favorable prognosis, exhibited the highest *HERC1* gene expression level, with a median value of 7.3 that was significantly higher than that of healthy controls. The recurrent CALR somatic mutations cause a +1 frameshift in the reading frame leading to mutant specific CALR carboxyl-terminus that lacks the Endoplasmic Reticulum (ER)-retention signal (KDEL). Calreticulin is a lectin chaperone that, alongside GRP78 and calnexin, promotes and facilitates the proper folding and maturation of the ER luminal proteins. Damaged or improperly folded proteins usually escape the calreticulin cycle and enter the ER Associated Degradation (ERAD). Otherwise proteins can continue their journey and when required move to Golgi Apparatus. Though CALR deficient cells exhibit accumulation of misfolded proteins, the ER structure remains unaltered. However, a significant increase in ubiquitin-proteasome activity has been detected as a result of a compensatory mechanism for cell survival [34]. Furthermore, it is well known that ER stress leads to activation of both the Unfolded Protein response (UPR) and autophagy processes that it turns out tightly depend on the ubiquitination process. A transcriptomic analysis carried out on a colon cancer cell line treated with ER stressors (e.g., brefeldin A and tunicamycin) revealed an up-regulation of a large group of autophagy related genes, including *HERC1* [35]. These data, together with the HERC1 subcellular localization in inner membranes (e.g., Golgi Apparatus) suggest that the Ubiquitin Proteasome System (UPS) might be a novel potential, and by now uninvestigated, player in the pathogenesis of the ET CALR mutation, and could contribute to the clonal advantage in hematopoietic cells by downregulating pro-apoptotic, or pro-differentiating, signaling processes. Furthermore, among the MPNs group those patients expressing either the highest, or the lowest, *HERC1* transcript levels define two novel subsets that overlap with ET CALR mutated and PMF groups thus leading to wonder whether the *HERC1* transcript levels might represent a novel additional diagnostic marker along with the ones currently available.

Differently from its closest relatives, namely ET, PV and PMF, the CML is characterized by a single genetic alteration giving rise to a constitutively active tyrosine kinase, the BCR-ABL1 fusion protein. This peculiar molecular landmark confers unique properties to be feasibly investigated from the very basic biological point of view by simply overexpressing, or pharmacologically inhibiting (e.g. Imatinib), the BCR-ABL1 protein. Our findings indicate that upon effective Imatinib treatment the *HERC1* mRNA levels raised sharply to levels similar to that of the control specimens but decreased again when the patients relapsed (Figure 4A). Consistently, cells overexpressing Bcr-Abl1 displayed lower *HERC1* transcript when compared to the control (Figure 5B–D). Though the genotype is different, the newly diagnosed AML, PMF and CML displayed low amounts of *HERC1* transcript when compared to the healthy control. Nonetheless, Bcr-Abl1 appears to trigger a decline in *HERC1* gene expression. Hence, it is conceivable to hypothesize that: (a) in AML, PMF and CML, HERC1 might act as an oncosuppressor [36] and (b) though genotypically different all these myeloid disorders share a common *HERC1* transcriptional repressor mechanism that in CML it is positively regulated by BCR-ABL1.

The different structural motifs characterizing the BCR-ABL1 fusion protein allow it to interact with dozens of binding partners [37]. Amongst them there are also few UPS components, comprising E3 Ubiquitin-ligases and deubiquitylating enzymes (e.g., Cbl, USP1) [38,39], that are fundamental to controlling the BCR-ABL1 stability and as a consequence its oncogenic potential. Our data indicate that HERC1 represents both a novel BCR-ABL1 interacting partner and a substrate. However, the functional meaning of the interaction and tyrosine phosphorylation remain presently to be ascertained. Currently, it cannot be excluded that HERC1 might act as BCR-ABL1 negative regulator and the tyrosine phosphorylation might control its enzymatic activity. Consistently, the enzymatic activity of Nedd4, a HECT E3 Ubiquitin-ligase member, is tightly regulated by the phosphorylation of a few tyrosine residues [40].

Additional studies are needed to dissect the functional relevance of the *HERC1* aberrant expression in myeloid related disorders. Indeed, though our data suggest that HERC1 plausibly plays a role as oncosuppressor in the majority of myeloid malignancies (e.g., AML, CML and PMF) by now we cannot rule out that in those ET calreticulin mutated it might display tumor-promoting properties, thus exerting different properties depending on the context. Determining the most relevant HERC1 substrates might represent a useful hint for novel druggable molecular targets.

## 4. Materials and Methods

### 4.1. Patients and Cell Lines

After written informed consent, specimens from CML, AML and MPNs were collected. In addition, peripheral blood (PB) and bone marrow (BM) specimens were also collected from healthy donors. Among CML samples, 37 were PB and 35 BM (Supplementary Table S1), among AML specimens, 80 were BM and 17 were PB (Supplementary Table S2) while MPNs specimens were PB (Supplementary Table S3). The K-562 and HEK-293T cell lines were cultured in complete RPMI-1640 and Dulbecco’s modified Eagle’s medium, respectively. Primary CML cells derived from patients’ bone marrow were cultured in ISCOVE’s medium, supplemented with heat inactivated 20% foetal bovine serum.

### 4.2. Quantitative Real-Time PCR

Total RNA was extracted, reverse transcribed and then transcript quantification analysis performed as described elsewhere [41]. For *HERC1* mRNA quantification, specific assays (assay ID for *GUSB* Hs00939627_m1, and Hs01032486_m1 for *HERC1*-Applied Biosystems, Thermo Fisher Scientific, Massachusetts, MA, USA) were used. The *HERC1* Ct values obtained by RT-qPCR were normalized with respect to the Ct counterparts of *GUSB* and expressed as 2^−∆∆Ct^. A universal human references RNA (Stratagene, San Diego, CA, USA) was used to calibrate the assay.

### 4.3. Cell Lysis and Immunoprecipitation

For total protein extraction, cells were washed with ice cold phosphate-buffered saline (PBS), and lysed as described [42]. For immunoprecipitation (IP) 1500 µg of total proteins were used. The protein lysates were incubated overnight at 4 °C with 2 µg of appropriate antibody and subsequently for 45 min at 4 °C with Protein A/G Sepharose (SC-2001#J1510, Santa Cruz Biotechnology). The immune-complex Sepharose beads were then washed with a lysis buffer and absorbed proteins were eluted in the SDS-sample buffer.

### 4.4. Western Blotting

One hundred micrograms (100 µg) of total protein were loaded and run onto 4%–14% gradient precasted gel (Criterion^TM^ TGX Stain-Free^TM^, Bio-Rad, Hercules, CA, USA) electrophoresis and transferred to PVDF (Bio-Rad, Hercules, CA, USA) membranes as described elsewhere [42]. Blots were blocked in TBS plus 5% non-fatty acid milk and decorated with appropriate primary antibodies (HERC1 #A301-904A, Bethyl Laboratories Inc., Montgomery, TX, USA; β-Actin, sc-47778, phospho-Tyr, sc-7020, c-Abl, sc-23 and BCR, sc-20707, Santa Cruz Biotechnology, Dallas, Texas, USA; Vinculin MA5-11690, Invitrogen, Carlsbad, CA, USA). Membranes were then washed and incubated with appropriate peroxidase-linked secondary antibody (Santa Cruz Biotechnology, Dallas, TX, USA). Specific binding was detected using an enhanced chemiluminescence system (Clarity Western ECL Substrate #170-5061, Bio-Rad, Hercules, CA, USA).

### 4.5. Immunofluorescence Staining

Cytospins were prepared using K562, BM and PB cells from CML patients, while HEK-293T cells were grown on coverslips [43]. Cells were fixed with 4% PFA, permeabilized with 0.1% TritonX100 and blocked with a blocking buffer (10% foetal bovine serum, 5% BSA, 1% fish gelatin). Afterwards, cells were washed with 1X-PBS and then incubated with anti-HERC1 antibody. Protein detection was obtained by incubation with a secondary antibody (A11011 Alexa Fluor 568 IgG, Invitrogen). Nuclear staining was performed with DAPI. Images were captured with a confocal microscope equipped with a 63x oil immersion objective (LSM 5110; Carl Zeiss. MicroImaging Inc., Oberkochen, Germany).

### 4.6. Plasmids and Transfection

The p210 *Bcr-Abl1* overexpressing plasmid [44] was used to transiently transfect HEK-293T cells by X-treme GeneHP (Roche, Basel, Switzerland) according to the manufacturer’s instructions.

### 4.7. In-Vitro Kinase Assay

The kinase assay was performed with immunoprecipitated HERC1 substrate and purified full-length c-ABL1 (Invitrogen, #P3049) as described elsewhere [44]. Briefly, the HEK-293T cells were lysed in CHAPS buffer (10 mM Tris–HCl pH 7.5, 100 mM NaCl, 0.3% CHAPS, 50 mM NaF, 0.5 mM EDTA, 1mM Na_3_VO_4_, 1mM PMSF, 2 µg/mL leupeptin, 5 µg/mL aprotinin, 2 µg/mL pepstatin), and endogenous HERC1 was immunoprecipitated as mentioned above in Section 4.3. Purified full-length ABL (Invitrogen, #P3049) and immunoprecipitated endogenous HERC1 were resuspended in kinase buffer (50 mM HEPES, 150 mM NaCl, 1 mM MgCl_2_, 1 mM MnCl_2_, 10 mM NaF, 1 mM NaVO_3_, 5% glycerol, 1% NP-40, 1 mM DTT, 1 mM PMSF) and 10 mM ATP (Sigma). After 30 min at 37 °C, reaction was stopped by the addition of SDS–PAGE buffer, followed by boiling for 10 min at 100 °C. Protein samples were separated on 4%-14% gradient precasted gel (Criterion^TM^ TGX Stain-Free^TM^, Bio-Rad, Hercules, CA, USA) electrophoresis and transferred to PVDF (Bio-Rad, Hercules, CA, USA) membranes, and incubated with anti-HERC1 and anti-phosphotyrosine antibodies. Membranes were then washed and incubated with appropriate peroxidase-linked secondary antibody (Santa Cruz Biotechnology, Dallas, Texas, USA). Specific binding was detected using an enhanced chemiluminescence system (Clarity Western ECL Substrate #170-5061, Bio-Rad, Hercules, CA, USA).

### 4.8. Statistical Analysis

Graphs are presented with error bars representing standard error calculation. Paired *t*-test or one-way ANOVA were used for non-parametric statistical analysis using GraphPad Prism v5.0 (GraphPad Software, Inc., La Jolla, CA, USA). * *p* < 0.05, ** *p* < 0.01, *** *p* < 0.001, **** *p* < 0.0001.

## 5. Conclusions

In conclusion, for the first time we assessed the expression levels of the gene encoding for the giant Hect E3 Ubiquitin ligase, HERC1, in white blood cells and in a panel of myeloid related disorders including AML, MPNs and CML. Though, in myeloid leukemia and related neoplasms, the *HERC1* transcript levels were mostly down-regulated, thus likely acting as an oncosuppressor, we noticed that there are few remarkable exceptions suggesting that its role is tightly context-dependent. The constitutively active BCR-ABL1 tyrosine kinase contributes significantly in lowering the *HERC1* gene expression. Into the bargain, the HERC1 protein represents a novel BCR-ABL1 interacting partner and substrate. Taken together our findings potentially open up a critical issue for further studies to learn more about HERC1 biology and its role in human diseases.

## Data Availability

We didn’t produce any public data base. There isn’t public data during this study.

## Supplementary Materials

The following are available online at https://www.mdpi.com/2072-6694/13/2/341/s1. Table S1: Chronic Myeloid Leukemia (CML) patient’s characteristics; Table S2: Characteristics of Acute Myeloid Leukemia (AML) patient’s at diagnosis; Table S3: Details of MyeloProliferative Neoplasms (MPNs) patients; Table S4: Age of AML patients enrolled in the study; Figure S1: The *HERC1* gene expression is differently regulated in healthy BM and PB; Figure S2: HERC1 is tyrosine phosphorylated in chronic myeloid leukemia K562 cells.
