# Supplementary material for: The Giant HECT E3 Ubiquitin Ligase HERC1 Is Aberrantly Expressed in Myeloid Related Disorders and It Is a Novel BCR-ABL1 Binding Partner
Source: Cancers (Basel). 2021 Jan 19;13(2):341. doi: 10.3390/cancers13020341 (PMC7832311; doi:10.3390/cancers13020341)
Supplement: Supplementary file 1 [file cancers-13-00341-s001.zip › Figure S1,Figure S2.pdf]

Figure S1

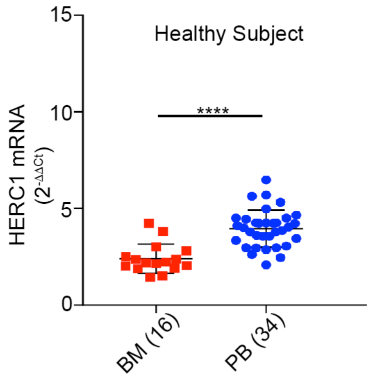

The *HERC1* gene expression is differently regulated in healthy BM and PB. The expression of the *HERC1* gene is differently regulated in healthy blood specimens. PB samples exhibited higher *HERC1* amount (median=4.0) when compared to the BM (median=2.2).

Figure S2

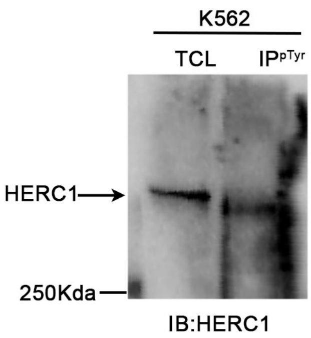

HERC1 is tyrosine phosphorylated in chronic myeloid leukemia K562 cells. K562 lysates were immunoprecipated by using anti phosphotyrosine (p-Tyr) antibody. Immunocomplex was subsequently resolved by SDS-PAGE, transferred onto PVDF membranes and eventually probed with HERC1 antibody. The presence of HERC1 band indicated that in K562 cells HERC1 is tyrosine-phosphorylated.
