# Supplementary material for: The Giant HECT E3 Ubiquitin Ligase HERC1 Is Aberrantly Expressed in Myeloid Related Disorders and It Is a Novel BCR-ABL1 Binding Partner
Source: Cancers (Basel). 2021 Jan 19;13(2):341. doi: 10.3390/cancers13020341 (PMC7832311; doi:10.3390/cancers13020341)
Supplement: Supplementary file 1 [file cancers-13-00341-s001.zip › supplementary 1,2,3,4 Tables_.pdf]

Table S1: Chronic Myeloid Leukemia (CML) patient's characteristics.

ID	SEX	Age	Disease	Type of Sample	Bcr-Abl1 (p210) Copy No (IS)
1	M	36	CML (Diagnosis)	Bone Marrow	61.203
2	F	35	CML (Diagnosis)	Bone Marrow	50.439
3	M	46	CML (Diagnosis)	Bone Marrow	74.213
4	M	82	CML (Diagnosis)	Bone Marrow	70.517
5	F	74	CML (Diagnosis)	Bone Marrow	40.876
6	M	65	CML (Diagnosis)	Bone Marrow	48.540
7	F	54	CML (Diagnosis)	Bone Marrow	55.957
8	F	76	CML (Diagnosis)	Bone Marrow	82.666
9	M	57	CML (Diagnosis)	Bone Marrow	79.062
10	M	55	CML (Diagnosis)	Bone Marrow	40.000
11	M	79	CML (Diagnosis)	Bone Marrow	119.340
12	M	52	CML (Diagnosis)	Bone Marrow	94.910
13	M	61	CML (Diagnosis)	Bone Marrow	88.418
14	M	61	CML (Diagnosis)	Bone Marrow	76.007
15	F	53	CML (Diagnosis)	Bone Marrow	54.700
16	F	66	CML (Diagnosis)	Bone Marrow	104.836
17	M	54	CML (Diagnosis)	Bone Marrow	157.280
18	M	11	CML (Diagnosis)	Bone Marrow	53.013
19	F	70	CML (Diagnosis)	Bone Marrow	94.800
20	F	52	CML (Diagnosis)	Bone Marrow	49.856
21	F	67	CML (Diagnosis)	Bone Marrow	100.477
22	M	79	CML (Diagnosis)	Bone Marrow	148.000
23	F	48	CML (Diagnosis)	Bone Marrow	100.980
24	F	63	CML (Diagnosis)	Bone Marrow	55.010
25	M	57	CML (Diagnosis)	Bone Marrow	68.500
26	F	53	CML (Diagnosis)	Bone Marrow	37.700
27	M	77	CML (Diagnosis)	Bone Marrow	40.061
28	F	56	CML (Diagnosis)	Bone Marrow	39.000
29	M	54	CML (Diagnosis)	Bone Marrow	59.020
30	F	61	CML (Diagnosis)	Bone Marrow	46.500
31	F	10	CML (Diagnosis)	Bone Marrow	15.600
32	F	68	CML (Diagnosis)	Bone Marrow	43.400
33	F	75	CML (Diagnosis)	Bone Marrow	87.000
34	F	72	CML (Diagnosis)	Bone Marrow	63.500
35	M	72	CML (Diagnosis)	Bone Marrow	68.630
36	M	63	CML (Diagnosis)	Peripheral Blood	39.223
37	F	58	CML (Diagnosis)	Peripheral Blood	52.139
38	M	71	CML (Diagnosis)	Peripheral Blood	65.898
39	F	85	CML (Diagnosis)	Peripheral Blood	30.821

40	M	48	CML (Diagnosis)	Peripheral Blood	74.544
41	M	82	CML (Diagnosis)	Peripheral Blood	97.660
42	F	74	CML (Diagnosis)	Peripheral Blood	51.951
43	F	76	CML (Diagnosis)	Peripheral Blood	80.899
44	M	20	CML (Diagnosis)	Peripheral Blood	26.395
45	M	15	CML (Diagnosis)	Peripheral Blood	135.310
46	M	56	CML (Diagnosis)	Peripheral Blood	85.735
47	M	57	CML (Diagnosis)	Peripheral Blood	80.013
48	F	57	CML (Diagnosis)	Peripheral Blood	42.000
49	F	26	CML (Diagnosis)	Peripheral Blood	44.000
50	M	66	CML (Diagnosis)	Peripheral Blood	59.972
51	M	79	CML (Diagnosis)	Peripheral Blood	158.590
52	M	52	CML (Diagnosis)	Peripheral Blood	112.800
53	M	61	CML (Diagnosis)	Peripheral Blood	100.000
54	M	61	CML (Diagnosis)	Peripheral Blood	72.772
55	M	54	CML (Diagnosis)	Peripheral Blood	137.000
56	M	11	CML (Diagnosis)	Peripheral Blood	57.729
57	F	70	CML (Diagnosis)	Peripheral Blood	193.500
58	M	45	CML (Diagnosis)	Peripheral Blood	58.001
59	F	52	CML (Diagnosis)	Peripheral Blood	48.335
60	M	37	CML (Diagnosis)	Peripheral Blood	53.736
61	M	67	CML (Diagnosis)	Peripheral Blood	98.500
62	M	79	CML (Diagnosis)	Peripheral Blood	93.350
63	F	48	CML (Diagnosis)	Peripheral Blood	102.350
64	M	54	CML (Diagnosis)	Peripheral Blood	57.200
65	F	74	CML (Diagnosis)	Peripheral Blood	48.100
66	M	71	CML (Diagnosis)	Peripheral Blood	70.500
67	F	72	CML (Diagnosis)	Peripheral Blood	82.970
68	M	50	CML (Diagnosis)	Peripheral Blood	93.900
69	M	36	CML (Diagnosis)	Peripheral Blood	38.200
70	f	83	CML (Diagnosis)	Peripheral Blood	79.000
71	M	54	CML (Diagnosis)	Peripheral Blood	86.200
72	M	77	CML (Diagnosis)	Peripheral Blood	74.130
73	F	40	CML (relapse)	Bone Marrow	12.979
74	M	76	CML (Resistant)	Bone Marrow	18.032
75	F	44	CML (relapse)	Bone Marrow	28.094
76	F	42	CML (Resistant)	Bone Marrow	25.597
77	F	31	CML (relapse)	Bone Marrow	14.383
78	F	79	CML (relapse)	Bone Marrow	58.691
79	F	83	CML (relapse)	Peripheral Blood	21.246
80	M	89	CML (relapse)	Peripheral Blood	47.862

81	M	79	CML Remission	Peripheral Blood	0.500
82	F	68	CML Remission	Peripheral Blood	0.030
83	M	38	CML Remission	Peripheral Blood	0.005
84	M	76	CML Remission	Peripheral Blood	0.022
85	M	69	CML Remission	Peripheral Blood	0.524
86	F	79	CML Remission	Peripheral Blood	0.144
87	F	61	CML Remission	Peripheral Blood	0.392
88	M	34	CML Remission	Peripheral Blood	0.015
89	M	35	CML Remission	Peripheral Blood	0.004
90	M	62	CML Remission	Peripheral Blood	0.000
91	M	18	CML Remission	Peripheral Blood	0.289
92	F	61	CML Remission	Peripheral Blood	0.056
93	F	32	CML Remission	Peripheral Blood	0.934
94	F	54	CML Remission	Peripheral Blood	0.035
95	M	64	CML Remission	Peripheral Blood	0.000
96	M	73	CML Remission	Peripheral Blood	0.008
97	M	38	CML Remission	Peripheral Blood	0.090
98	F	68	CML Remission	Peripheral Blood	0.002
99	F	54	CML Remission	Peripheral Blood	2.900
100	M	48	CML Remission	Peripheral Blood	0.070
101	F	36	CML Remission	Peripheral Blood	0.000
102	F	70	CML Remission	Peripheral Blood	0.280
103	F	54	CML Remission	Peripheral Blood	0.035
104	F	44	CML Remission	Peripheral Blood	0.025
105	M	57	CML Remission	Peripheral Blood	0.980
106	F	65	CML Remission	Peripheral Blood	0.006
107	M	80	CML Remission	Peripheral Blood	0.080
108	M	12	CML Remission	Peripheral Blood	0.010
109	F	63	CML Remission	Peripheral Blood	1.000
110	F	66	CML Remission	Peripheral Blood	0.700
111	F	60	CML Remission	Peripheral Blood	0.000
112	F	39	CML Remission	Bone Marrow	1.072
113	M	49	CML Remission	Bone Marrow	0.060
114	F	54	CML Remission	Peripheral Blood	0.020
115	M	62	CML Remission	Peripheral Blood	0.060
116	F	58	CML Remission	Peripheral Blood	0.081
117	M	57	CML Remission	Peripheral Blood	0.008
118	F	20	CML Remission	Peripheral Blood	0.100
119	F	75	CML Remission	Peripheral Blood	0.000
120	F	74	CML Remission	Peripheral Blood	0.057
121	M	39	CML Remission	Peripheral Blood	1.250

122	f	87	CML Remission	Peripheral Blood	0.000
123	M	56	CML Remission	Peripheral Blood	0.093
124	M	61	CML Remission	Peripheral Blood	0.000
125	F	54	CML Remission	Peripheral Blood	0.280

Table S2: Characteristics of Acute Myeloid Leukemia (AML) patient's at diagnosis.

ID	Sex	Age	Disease	Type of Sample	Genetic Alteration
1	F	58	AML (Diagnosis)	Bone Marrow	inv16
2	M	63	AML (Diagnosis)	Bone Marrow	inv16
3	m	38	AML (Diagnosis)	Bone Marrow	inv16
4	M	6	AML (Diagnosis)	Bone Marrow	inv16
5	M	71	AML (Diagnosis)	Bone Marrow	inv16
6	M	42	AML (Diagnosis)	Bone Marrow	inv16
7	F	57	AML (Diagnosis)	Bone Marrow	inv16
8	M	64	AML (Diagnosis)	Bone Marrow	inv16
9	F	58	AML (Diagnosis)	Bone Marrow	inv16
10	M	64	AML (Diagnosis)	Bone Marrow	t (8;21)
11	M	51	AML (Diagnosis)	Bone Marrow	t (8;21)
12	M	38	AML (Diagnosis)	Bone Marrow	t (8;21)
13	M	33	AML (Diagnosis)	Bone Marrow	t (8;21)
14	M	29	AML (Diagnosis)	Bone Marrow	t (8;21)
15	M	63	AML (Diagnosis)	Bone Marrow	t (8;21)
16	M	34	AML (Diagnosis)	Bone Marrow	t(8;21)
17	M	75	AML (Diagnosis)	Bone Marrow	t (8;21)
18	M	31	AML (Diagnosis)	Bone Marrow	t (8;21)
19	M	73	AML (Diagnosis)	Bone Marrow	IDH2 R140Q
20	M	55	AML (Diagnosis)	Bone Marrow	IDH2 R140Q
21	m	84	AML (Diagnosis)	Bone Marrow	IDH2 R140Q
22	F	82	AML (Diagnosis)	Bone Marrow	IDH2 R140Q
23	M	79	AML (Diagnosis)	Bone Marrow	IDH2 R140Q
24	M	57	AML (Diagnosis)	Bone Marrow	NPM1
25	F	78	AML (Diagnosis)	Bone Marrow	NPM1
26	M	64	AML (Diagnosis)	Bone Marrow	NPM1
27	M	74	AML (Diagnosis)	Bone Marrow	NPM1
28	F	76	AML (Diagnosis)	Bone Marrow	NPM1
29	F	22	AML (Diagnosis)	Bone Marrow	NPM1
30	F	69	AML (Diagnosis)	Bone Marrow	NPM1
31	F	51	AML (Diagnosis)	Bone Marrow	NPM1
32	M	91	AML (Diagnosis)	Bone Marrow	FLT3 ITD
33	M	75	AML (Diagnosis)	Bone Marrow	FLT3 ITD
34	F	67	AML (Diagnosis)	Bone Marrow	FLT-3 15% COD. 835

35	F	77	AML (Diagnosis)	Bone Marrow	FLT-3 ITD
36	F	53	AML (Diagnosis)	Bone Marrow	FLT-3 ITD
37	M	36	AML (Diagnosis)	Bone Marrow	FLT-3 ITD
38	M	48	AML (Diagnosis)	Bone Marrow	FLT-3 ITD
39	M	41	AML (Diagnosis)	Bone Marrow	FLT-3 ITD
40	M	59	AML (Diagnosis)	Bone Marrow	FLT-3 ITD
41	F	58	AML (Diagnosis)	Bone Marrow	FLT-3 ITD/inv16
42	M	41	AML (Diagnosis)	Bone Marrow	FLT-3 ITD/t(15;17)
43	F	81	AML (Diagnosis)	Bone Marrow	NPM1-FLT3 ITD
44	F	88	AML (Diagnosis)	Bone Marrow	NPM1-FLT3 ITD
45	F	52	AML (Diagnosis)	Bone Marrow	FLT3 ITD
46	M	46	AML (Diagnosis)	Bone Marrow	FLT3 ITD
47	M	47	AML (Diagnosis)	Bone Marrow	FLT3 ITD
48	F	38	AML (Diagnosis)	Bone Marrow	No
49	M	56	AML (Diagnosis)	Bone Marrow	No
50	M	18	AML (Diagnosis)	Bone Marrow	No
51	F	66	AML (Diagnosis)	Bone Marrow	No
52	M	73	AML (Diagnosis)	Bone Marrow	No
53	F	82	AML (Diagnosis)	Bone Marrow	No
54	F	78	AML (Diagnosis)	Bone Marrow	No
55	M	30	AML (Diagnosis)	Bone Marrow	No
56	M	72	AML (Diagnosis)	Bone Marrow	No
57	M	57	AML (Diagnosis)	Bone Marrow	No
58	M	35	AML (Diagnosis)	Bone Marrow	No
59	M	65	AML (Diagnosis)	Bone Marrow	No
60	M	40	AML (Diagnosis)	Bone Marrow	No
61	F	61	AML (Diagnosis)	Bone Marrow	No
62	M	76	AML (Diagnosis)	Bone Marrow	No
63	F	68	AML (Diagnosis)	Bone Marrow	t(15;17)PML/RARA
64	F	64	AML (Diagnosis)	Bone Marrow	t(15;17)PML/RARA
65	M	68	AML (Diagnosis)	Bone Marrow	t(15;17)PML/RARA
66	F	79	AML (Diagnosis)	Bone Marrow	t(15;17)PML/RARA
67	M	41	AML (Diagnosis)	Bone Marrow	FLT-3 ITD/t(15;17)
68	M	41	AML (Diagnosis)	Bone Marrow	t(15;17)PML/RARA
69	F	79	AML (Diagnosis)	Bone Marrow	t(15;17)PML/RARA
70	F	34	AML (Diagnosis)	Bone Marrow	t(15;17)PML/RARA
71	M	85	AML (Diagnosis)	Bone Marrow	t(15;17)PML/RARA
72	F	59	AML (Diagnosis)	Bone Marrow	t(15;17)PML/RARA
73	M	56	AML (Diagnosis)	Bone Marrow	t(15;17)PML/RARA
74	M	38	AML (Diagnosis)	Bone Marrow	t(15;17)PML/RARA
75	M	74	AML (Diagnosis)	Bone Marrow	t(15;17)PML/RARA
76	F	40	AML (Diagnosis)	Bone Marrow	t(15;17)PML/RARA
77	F	79	AML (Diagnosis)	Bone Marrow	NPM1-FLT3 835
78	F	70	AML (Diagnosis)	Bone Marrow	NPM1-FLT3 835
79	M	68	AML (Diagnosis)	Bone Marrow	NPM1-FLT3 835
80	M	76	AML (Diagnosis)	Bone Marrow	NPM1-FLT3 835

81	M	79	AML (Diagnosis)	Peripheral Blood	inv16
82	M	51	AML (Diagnosis)	Peripheral Blood	inv16
83	m	57	AML (Diagnosis)	Peripheral Blood	inv16
84	F	34	AML (Diagnosis)	Peripheral Blood	inv16
85	F	87	AML (Diagnosis)	Peripheral Blood	t(8;21)
86	F	51	AML (Diagnosis)	Peripheral Blood	t(8;21)
87	M	55	AML (Diagnosis)	Peripheral Blood	IDH2 R140Q
88	F	51	AML (Diagnosis)	Peripheral Blood	NPM1
89	F	24	AML (Diagnosis)	Peripheral Blood	NPM1
90	F	51	AML (Diagnosis)	Peripheral Blood	FLT-3 ITD/t(15;17)
91	F	80	AML (Diagnosis)	Peripheral Blood	FLT-3 ITD
92	F	51	AML (Diagnosis)	Peripheral Blood	FLT-3 ITD/t(15;17)
93	M	55	AML (Diagnosis)	Peripheral Blood	IDH2 R140Q
94	F	88	AML (Diagnosis)	Peripheral Blood	NPM1-FLT3 835
95	F	51	AML (Diagnosis)	Peripheral Blood	FLT-3 ITD
96	F	75	AML (Diagnosis)	Peripheral Blood	IDH2 & IDH1
97	M	37	AML (Diagnosis)	Peripheral Blood	t(8;21)

Table S3: Details of MyeloProliferative Neoplasms (MPNs) patients.

ID	Sex	Age (aa)	Disease	BM/PB	Mutation
1	M	87	ET (Diagnosis)	PB	CARL-type 2
2	F	53	ET (Diagnosis)	PB	CARL-type 1
3	F	68	ET (Diagnosis)	PB	CARL-type 1
4	M	57	ET (Diagnosis)	PB	CARL-type 1
5	M	55	ET (Diagnosis)	PB	CARL-type 1
6	M	53	ET (Diagnosis)	PB	CARL-type 1
7	F	70	ET (Diagnosis)	PB	CARL-type 1
8	M	59	ET (Diagnosis)	PB	CARL-type 1
9	M	48	ET (Diagnosis)	PB	CARL-type 9
10	M	64	ET (Diagnosis)	PB	CARL-type 1
11	M	47	ET (Diagnosis)	PB	CARL-type 2
12	M	50	ET (Diagnosis)	PB	CARL-type 4
13	M	83	ET (Diagnosis)	PB	CARL-type 1
14	M	62	ET (Diagnosis)	PB	CARL-type 2
15	F	54	ET (Diagnosis)	PB	CARL-type 2
16	F	57	ET (Diagnosis)	PB	CARL-type 1
17	F	82	ET (Diagnosis)	PB	$JAK2^{V617F}$
18	M	47	ET (Diagnosis)	PB	$JAK2^{V617F}$
19	M	78	ET (Diagnosis)	PB	$JAK2^{V617F}$
20	F	49	ET (Diagnosis)	PB	$JAK2^{V617F}$

21	M	63	ET (Diagnosis)	PB	$JAK2^{V617F}$
22	M	19	ET (Diagnosis)	PB	$JAK2^{V617F}$
23	F	65	ET (Diagnosis)	PB	$JAK2^{V617F}$
24	F	59	ET (Diagnosis)	PB	w.t.
25	F	42	ET (Diagnosis)	PB	NR
26	F	82	ET (Diagnosis)	PB	w.t.
27	M	59	ET (Diagnosis)	PB	w.t.
28	F	70	ET (Diagnosis)	PB	w.t.
29	F	75	ET (Diagnosis)	PB	w.t.
30	M	57	ET (Diagnosis)	PB	w.t.
31	M	64	ET (Diagnosis)	PB	w.t.
32	M	53	ET (Diagnosis)	PB	w.t.
33	M	75	ET (Diagnosis)	PB	w.t.
34	M	51	ET (Diagnosis)	PB	w.t.
35	F	52	ET (Diagnosis)	PB	NR
36	F	70	ET (Diagnosis)	PB	w.t.
37	F	27	ET (Diagnosis)	PB	w.t.
38	F	55	ET (Diagnosis)	PB	NR
39	M	92	ET (Diagnosis)	PB	w.t.
40	M	77	PV (Diagnosis)	PB	$JAK2^{V617F}$
41	M	49	PV (Diagnosis)	PB	$JAK2^{V617F}$
42	M	84	PV (Diagnosis)	PB	$JAK2^{V617F}$
43	F	37	PV (Diagnosis)	PB	$JAK2^{V617F}$
44	F	77	PV (Diagnosis)	PB	$JAK2^{V617F}$
45	M	56	PV (Diagnosis)	PB	$JAK2^{V617F}$
46	F	89	PV (Diagnosis)	PB	$JAK2^{V617F}$
47	F	60	PV (Diagnosis)	PB	$JAK2^{V617F}$
48	F	84	PV (Diagnosis)	PB	$JAK2^{V617F}$
49	S	38	PV (Diagnosis)	PB	$JAK2^{V617F}$
50	M	69	PV (Diagnosis)	PB	$JAK2^{V617F}$
51	M	69	PV (Diagnosis)	PB	w.t.
52	M	64	PV (Diagnosis)	PB	w.t.
53	M	45	PV (Diagnosis)	PB	w.t.
54	M	58	PV (Diagnosis)	PB	w.t.
55	M	66	PV (Diagnosis)	PB	w.t.
56	M	28	PV (Diagnosis)	PB	w.t.
57	M	72	PV (Diagnosis)	PB	w.t.
58	M	74	PV (Diagnosis)	PB	w.t.
59	M	65	PV (Diagnosis)	PB	w.t.
60	M	47	PV (Diagnosis)	PB	w.t.
61	M	80	PMF (Diagnosis)	PB	$JAK2^{V617F}$
62	M	69	PMF (Diagnosis)	PB	$JAK2^{V617F}$
63	F	85	PMF (Diagnosis)	PB	$JAK2^{V617F}$
64	M	78	PMF (Diagnosis)	PB	$JAK2^{V617F}$

65	M	60	PMF (Diagnosis)	PB	$JAK2^{V617F}$
66	F	76	PMF (Diagnosis)	PB	$JAK2^{V617F}$
67	F	67	PMF (Diagnosis)	PB	$JAK2^{V617F}$
68	M	77	PMF (Diagnosis)	PB	w.t.
69	F	84	PMF (Diagnosis)	PB	NR
70	M	42	PMF (Diagnosis)	PB	w.t.
71	M	39	PMF (Diagnosis)	PB	w.t.
72	M	47	PMF (Diagnosis)	PB	w.t.
73	F	60	PMF (Diagnosis)	PB	w.t.
74	M	40	PMF (Diagnosis)	PB	w.t.
75	F	73	PMF (Diagnosis)	PB	w.t.
76	F	78	PMF (Diagnosis)	PB	w.t.
77	F	78	PMF (Diagnosis)	PB	w.t.
78	F	68	PMF (Diagnosis)	PB	w.t.

Table S4: (expressed either as median value and as range) of the AML patients enrolled in the study. ***ITD:*** Internal Tandem Duplication; ***y:*** years; ***n:*** number of patients

**Mutation free (n=15)**	**NPM-FT3 D835 (n=5)**	**t(15;17) (n=14)**	**IDH2 R^{140Q} (n=5)**	**t(8;21) (n=9)**	**FLT3 ITD (n=14)**	**inv16 (n=9)**	**NPM1 (n=10)**
Median: 68 y<br>***Range:*** 23÷83	***Median***: 75 y<br>***Range:*** 51÷90	***Median:*** 67 y<br>***Range:*** 39÷84	***Median:*** 64 y<br>***Range:*** 64÷75	***Median:*** 54 y<br>***Range:*** 36÷89	***Median:*** 58 y<br>***Range***: 41÷92	***Median:*** 60 y<br>***Range:*** 45÷63	***Median:*** 55 y<br>***Range:*** 26÷68
